# Effects of Internet-Based Self-Efficacy Intervention on Secondary Traumatic Stress and Secondary Posttraumatic Growth among Health and Human Services Professionals Exposed to Indirect Trauma

**DOI:** 10.3389/fpsyg.2016.01009

**Published:** 2016-07-04

**Authors:** Roman Cieslak, Charles C. Benight, Anna Rogala, Ewelina Smoktunowicz, Martyna Kowalska, Katarzyna Zukowska, Carolyn Yeager, Aleksandra Luszczynska

**Affiliations:** ^1^Department of Psychology, SWPS University of Social Sciences and Humanities, WarsawPoland; ^2^Trauma Health and Hazards Center, Department of Psychology, University of Colorado Colorado Springs, Colorado Springs, COUSA; ^3^Wroclaw Department, SWPS University of Social Sciences and Humanities, WroclawPoland

**Keywords:** secondary traumatic stress, posttraumatic growth, internet-based intervention, self-efficacy, work stress

## Abstract

**Background:** Although the evidence for the associations among self-efficacy, secondary traumatic stress (STS) and secondary posttraumatic growth (SPTG) is mounting, there is a lack of the experimental evidence for the influence of self-efficacy on positive and negative mental health outcomes among professionals indirectly exposed to trauma.

**Purpose:** This study investigated the effects of an internet-based self-efficacy intervention (the experimental condition), compared to an education (the active control condition) on STS and SPTG among workers exposed to traumatic events indirectly, through their clients. We hypothesized that the group assignment (experimental vs. control) would affect STS and SPTG indirectly, with a mediating role of self-efficacy beliefs.

**Methods:** Participants were 168 health and human services professionals (78% women), exposed indirectly to a traumatic event at work. They were randomly assigned to either a 4-session internet-based self-efficacy intervention (*n* = 87) or an education control group (*n* = 81) which received information about coping resources and consequences of stressors at work or at home. STS, SPTG, and self-efficacy were measured at the baseline (Time 1), 1-month follow-up (Time 2) and 2-month follow-up (Time 3).

**Results:** Analysis of covariance showed that the group assignment had a significant effect on STS (Time 2) and self-efficacy (Time 2), with lower STS and higher self-efficacy reported by the self-efficacy intervention participants. Compared to the experimental group, the active control (education) group participants reported higher SPTG at Time 2. Mediation analyses indicated that the group assignment had indirect effects on STS and SPTG at Time 3. Workers who experienced increases in self-efficacy (Time 2) through the intervention were more likely to report lower STS and higher SPTG at Time 3.

**Conclusion:** Elucidating the mediating processes that explain why an intervention for secondary trauma works is essential in order to develop more effective support systems that promote improved mental health outcomes among health and human services professionals. Prevention programs for workers exposed indirectly to traumatic events may target self-efficacy enhancement and education.

## Introduction

The quality of work and health among human service professionals dealing with traumatized populations depends on their ability to manage stress at work and, consequently, their mental health and well-being (Killian, 2008). The last two decades has brought an increase of interest in one type of work-related stressor that may influence the mental health of human service workers, namely indirect exposure to trauma through work (Pearlman and Mac Ian, 1995; Voss Horrell et al., 2011). The effects of indirect (also called vicarious or secondary) exposure to trauma through work with traumatized patients or clients on the mental health of human service professionals are well-recognized (Voss Horrell et al., 2011; Cohen and Collens, 2013). On one hand, indirect exposure might have a positive effect on providers’ posttraumatic growth (Cohen and Collens, 2013). On the other hand, it is also predictive of higher distress (Pearlman and Mac Ian, 1995), increased negative cognitions (Pearlman and Mac Ian, 1995), and higher risk of job burnout (Cieslak et al., 2014). Organizational factors and individual variables protecting workers from negative effects of indirect exposure and enhancing potentially positive effects of exposure were thoroughly studied (for reviews see Voss Horrell et al., 2011; Cohen and Collens, 2013). However, the effectiveness and mechanisms of psychosocial interventions for reducing the negative outcomes and enhancing the positive effects of indirect exposure at work have been neglected. To fill this void, the present study offers insight into the effects and mechanisms of a psychosocial intervention which aims at enhancing secondary posttraumatic growth (SPTG) and reducing secondary posttraumatic stress among health and human services professionals.

Indirect exposure takes place when workers are exposed to traumatic content in their professional contacts with traumatic stress survivors (cf. Elwood et al., 2011). Secondary traumatic stress (STS) is one of the mental health consequences specific to the indirect exposure to trauma (Bride et al., 2004). STS may be manifested by symptoms which are similar to posttraumatic stress disorder symptoms of re-experiencing, avoidance, and hyperarousal (Figley, 1995; Elwood et al., 2011). Recent meta-analyses suggested that various aspects of indirect exposure, including its frequency and volume, were related with STS symptoms across health and human services professions (Hensel et al., 2015).

Due to indirect exposure to traumatic events, health and human service professionals may be at risk of developing STS. Professions that are at high risk of developing STS include social workers (Bride, 2007) child protective services workers (Bride et al., 2007), military health providers (Voss Horrell et al., 2011), civilian healthcare providers (Sheen et al., 2014), and clinicians working with trauma survivors (Elwood et al., 2011). Professionals who are at risk for indirect exposure to traumatic events, and, therefore, at risk for STS are in need of brief interventions protecting their mental health. Addressing the concept of STS in interventions targeting health service professionals may help to de-stigmatize their mental health problems and reinforce the need for training and preventative care (Royle et al., 2009).

Besides the negative consequences of exposure to trauma, recent research indicated that there are also positive changes following exposure to trauma, such as meaning making (Park, 2010) and posttraumatic growth (Cann et al., 2010). Recent meta-analyses indicated that psychosocial interventions significantly affect posttraumatic growth among trauma survivors (Roepke, 2015). Building on the posttraumatic growth construct, Arnold et al. (2005) coined the term ‘vicarious posttraumatic growth,’ referring to positive changes in schemas about self and the world and perceived psychological growth. In the context of health and human services personnel, the terms vicarious and secondary are used interchangeably to denote posttraumatic growth resulting from working with traumatized clients (cf. Shoji et al., 2015).

Secondary posttraumatic growth is one of the key outcomes of indirect exposure to trauma experienced by health and human service professionals (Cohen and Collens, 2013). It results from being exposed to and shocked with materials revealed by traumatized clients and witnessing client’s strength and growth during the processes of dealing with their traumatic experiences (Cohen and Collens, 2013). According to the SPTG model proposed by Cohen and Collens (2013), the areas of SPTG include changes in beliefs about world (e.g., appreciation of life and human resilience), values (importance of support from family), self (increasing self-awareness and beliefs about one’s own worth and capability), and daily life (e.g., actively reacting to social problems of others). Furthermore, research suggested that STS and SPTG may be unrelated, but they may operate jointly in explaining other mental health problems, such as depression, anxiety, finding meaning, and satisfaction with life (Samios et al., 2012). Thus, STS and SPTG should be considered as key outcomes of indirect exposure, which may further contribute to other mental health outcomes.

Originally, the concept of self-efficacy was developed in the context of dealing with various stressful events, barriers and obstacles preventing individuals from actively influencing their own inner states and the environment (Bandura, 1997). However, self-efficacy and social cognitive theory were adopted to explain processes of adaptation to traumatic events (Benight and Bandura, 2004). Self-efficacy refers to an individual’s beliefs about their own ability to cope with demands (including those referring to work-related stressors). Those beliefs enable individuals to deal more effectively with stressors (including traumatic events) and promote health and well-being (Bandura, 1997).

Systematic reviews indicated that among trauma survivors, self-efficacy is related to better mental and physical health (Luszczynska et al., 2009). Research conducted among healthcare workers indicated that self-efficacy predicts better mental health outcomes and higher SPTG (Shoji et al., 2014; Rogala et al., 2015). Furthermore, SPTG refers to positive changes or enhancements in such areas as personal activism or agency, and personal capabilities (Cohen and Collens, 2013), which may partially overlap with the core aspects of self-efficacy (Bandura, 1997).

Self-efficacy is a modifiable cognition, which may be enhanced by a mastery experience and reinforced by positive affective states (Bandura, 1997). Therefore, interventions promoting mental and physical health often used self-efficacy enhancing techniques (Luszczynska and Schwarzer, 2015). Yet, those interventions were rarely applied in the context of traumatic stress.

So far, the effects of internet-based interventions enhancing self-efficacy beliefs were investigated in the context of the direct exposure to traumatic events (Steinmetz et al., 2012). A brief self-efficacy intervention developed by Steinmetz et al. (2012) targeted self-efficacy through mastery experience, verbal persuasion, and emotion self-regulation techniques. It also provided tools enabling survivors to seek social support for dealing with consequences of exposure to a natural disaster. The effects of this self-efficacy intervention were compared with an education (information-only) control condition and with the ‘usual care’ (e.g., suggesting to seek for mental health consultations, if needed) within a group of 56 survivors of Hurricane Ike. Importantly, both control conditions were passive (read-only) and did not include any interactive exercises. Although PTSD symptoms were unaffected, a significant reduction (or a trend for a reduction) was found for worry and depression (Steinmetz et al., 2012). To our knowledge, there are no studies that demonstrate whether a manipulation aimed at a change in self-efficacy beliefs may affect both positive and negative mental health outcomes which occur in the context of indirect exposure to traumatic events. The present study aims at filling this void.

Psychosocial interventions aiming at STS prevention and promotion of SPTG may focus on educating professionals to enable them to cope with stressful events at work (Newell and MacNeil, 2010). Newell and MacNeil (2010) proposed a best-practice initiative, suggesting thorough education programs for workers indirectly exposed to trauma. As a part of compulsory training for professionals, this education should be delivered to both experienced and inexperienced professionals and include information about indirect exposure, its consequences and preventative resources (Newell and MacNeil, 2010). Thus, the education programs may be considered an alternative to interventions enhancing beliefs such as self-efficacy. Unfortunately, the investigation whether such education interventions affect mental health outcomes (e.g., STS or SPTG) is missing.

There is a growing interest in using internet-based interventions to reduce negative consequences of stress at work (Shimazu et al., 2005; Cook et al., 2007). Internet-based interventions were frequently applied in programs aimed at the reduction of posttraumatic stress disorder (PTSD) among survivors of direct exposure to traumatic events (Kuester et al., 2016). However, the effects of internet-based interventions targeting people exposed to indirect trauma (through their work) were not evaluated.

The effectiveness of interventions is evaluated by comparing their influence on chosen outcomes to the influence exerted by specific control groups. The control groups may differ regarding the content (e.g., education or cognitive-behavioral techniques) and procedures (i.e., passive, ‘read-only’ procedures versus active, with interactive exercises). The specificity of the content and procedures applied in the control group has to be accounted for when interpreting the findings. For example, a recent meta-analysis evaluating PTSD symptoms among survivors of direct exposure to trauma (Kuester et al., 2016) compared the effects of internet-based interventions (active procedures; cognitive-behavioral techniques) to (1) the control conditions with no treatment at all (e.g., waiting list) and (2) other control conditions (e.g., passive or active procedures; education or internet-based writing exercises but no specific cognitive-behavioral techniques). Internet-based interventions were effective in reducing PTSD when their influence was compared with the effects of participation in control groups with no treatment at all. However, the effects of the internet-based interventions on PTSD were similar to effects found for psychoeducation or other control conditions, (cf. Kuester et al., 2016). Thus, the content and the procedures used in the control conditions need to be accounted for in future research.

It may be assumed that interactive intervention procedures with exercises that require recalling and writing about one’s own experiences may constitute mastery experiences. Mastery experiences represent the main source of self-efficacy (Bandura, 1997; Luszczynska and Schwarzer, 2015). Therefore, the interactivity of the self-efficacy enhancing intervention may be a condition necessary for its effectiveness. Previous research showed that self-efficacy interventions with interactive procedures requiring recalling and reporting one’s own experiences were more effective than passive (read-only) control procedures which focus on education (Steinmetz et al., 2012; Luszczynska et al., 2016). Therefore, the present study compared the interactive self-efficacy intervention with a read-only education condition.

Besides knowing *if* the intervention works, it is crucial to know *how* it works. The evaluation of underlying mechanisms may be achieved using a mediation analysis, testing whether the assignment to the experimental condition explains the outcome variables indirectly through a change in the psychological variables matched to the intervention techniques. Therefore, to prove that a self-efficacy intervention affected STS or SPTG, studies need to show that these effects are actually mediated by self-efficacy beliefs boosted by the experimental manipulation. Unfortunately, although testing for the underlying changes in self-efficacy became a standard in research on health behavior (cf. Luszczynska and Schwarzer, 2015; Luszczynska et al., 2016), research investigating mental health promoting interventions rarely provided explicit tests of the underlying mediating mechanisms. Without specifying and testing for the underlying mechanisms, even a well-designed study cannot be informative of *how* an intervention worked (Abraham et al., 2014).

This study aimed at evaluating the influence of the self-efficacy enhancing intervention on STS and SPTG among health and human services workers exposed indirectly to traumatic events. The effects of the self-efficacy enhancing experimental condition were compared to a control (education) condition. Furthermore, the study investigated the underlying mechanisms, specifying that the effects of the intervention may be explained by its influence on self-efficacy.

In particular, it was hypothesized that compared to the control (read-only; education) condition, participants in the experimental (interactive; self-efficacy) condition would present lower STS and higher SPTG at 1-month follow-up (Time 2) and at 2-month follow-up (Time 3). Second, it was hypothesized that the effects of the group assignment (control vs. experimental) would indirectly influence STS and SPTG at 2-month follow-up (Time 3), with self-efficacy at Time 2 playing the mediating role. These effects were expected to occur after controlling for the values of self-efficacy and the respective outcome variable (either STS or SPTG) at the baseline (Time 1).

## Materials and Methods

### Participants

Participants were 168 health and human services professionals, exposed indirectly to traumatic events at work. On average, they were 37.49 years old (*SD* = 10.39), and the majority of them were women (78%). The sample consisted of healthcare providers (physicians, nurses, first responders; 29.8%), social workers (21.4%), psychotherapists (13.7%), education specialists (teachers, counselors; 24.4%), police officers and firefighters (3.0%), and other human service providers (6.5%). They were employed from 1 to 32 years, with the mean of 8.53 (*SD* = 8.24) years.

Participants were recruited via advertisements in newspapers, internet forums, and websites dedicated to respective professionals, and through professional organizations in Poland. The recruitment took place between October 2012 and May 2013. Those who were interested in participating in the study filled out the contact forms and then received information about the study aims and procedures. All respondents provided the informed consent. Those who gave the informed consent received a link to an online screening questionnaire.

The initial screening aimed at identifying professionals who met the inclusion criteria which included providing services for survivors of traumatic events for at least 1 year, experiencing indirect exposure to a traumatic event at work, and consent for participating in an internet-based program aiming at the enhancement of psychosocial resources improving mental health.

A total of 265 professionals expressed preliminary interest in participating, filled in contact forms, and provided informed consent. In the next step, we excluded 97 potential respondents who either did not meet the inclusion criterion, that is experiencing indirect exposure to trauma at work (*n* = 69) or/and they declined to participate (*n* = 28).

**FIGURE 1:**
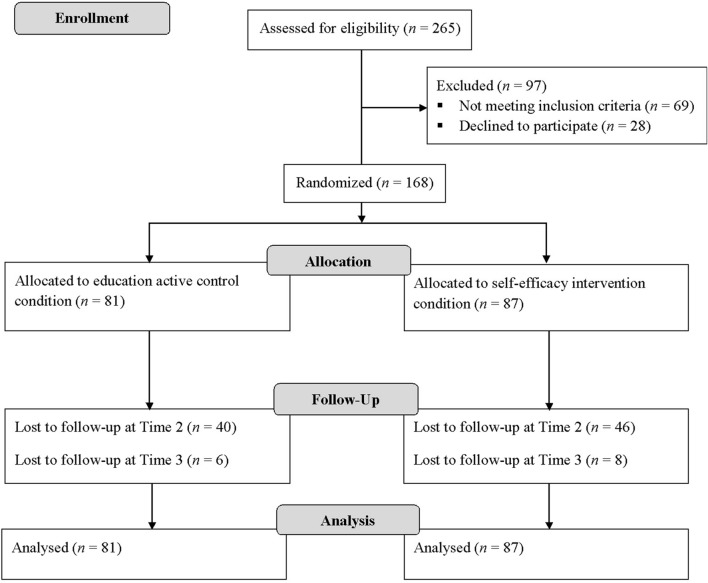
**Flow of participants in a study**.

Data from 168 participants were analyzed. Respondents were randomly assigned to the experimental and control groups: the self-efficacy enhancement intervention (*n* = 87) or an education active control group (*n* = 81). Only 68 participants completed all experimental/control group procedures and participated in the measurements at T1, T2, and T3. Overall, 54 participants dropped out from the experimental condition, and 46 dropped out from the control condition, making a total of 100 (59.5%). **Figure 1** presents the participant flow across the stages of data collection.

### Methodology

Data were collected three times: at the baseline (Time 1), at the 1-month follow up (Time 2; after completing the intervention/control group procedures), and at the 2-month follow-up (Time 3; 2 months after the baseline). Automatic emails were sent as a reminder to complete the measurements: 1 day after the scheduled Time 1 measurement, 3 days after the scheduled Time 2 measurement, and 3 days after the Time 3 measurement. The study was approved by the Institutional Review Board at the SWPS University of Social Sciences and Humanities.

The experimental and control group procedures were delivered via a designated website. They consisted of four sessions that included: (1) introductory informational materials, (2) self-efficacy exercises or extended information materials in the experimental and control groups respectively, (3) homework assignments, and (4) summaries of the session. The intervention and control group procedures were delivered over 4 weeks.

Participants of the control and intervention groups were asked to make notes in their web-based personal diary to keep track of their thoughts referring to the sessions and their content. All respondents were provided with an option to ask the experimenters about the technical and procedural issues referring to the sessions. All experimenters had a Master’s degree in psychology and had at least 1 year work experience in the context of occupational health.

**FIGURE 2:**
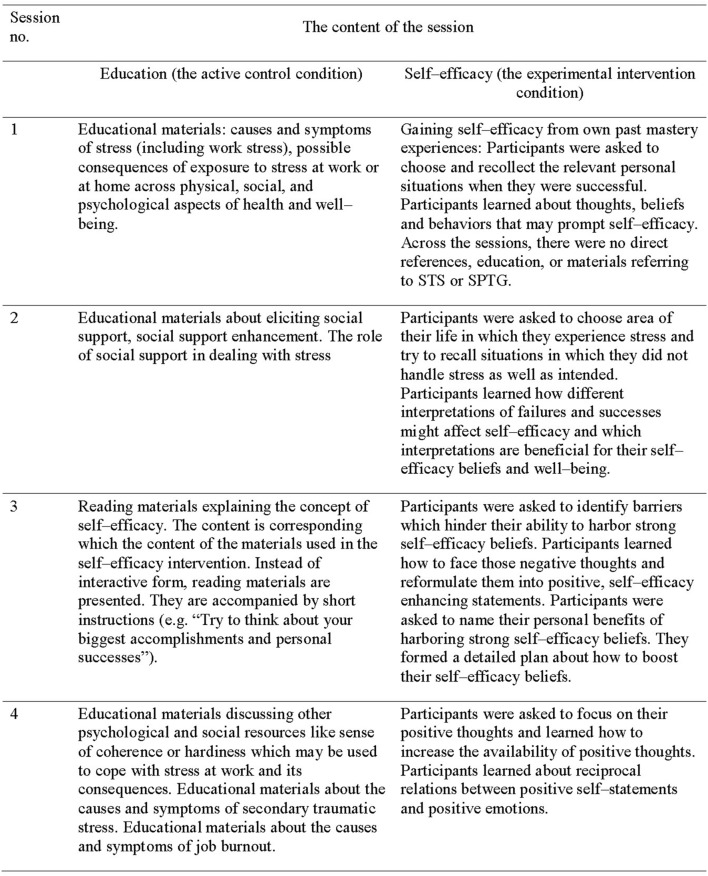
**The content of self-efficacy intervention and active control education condition**.

Automatic email reminders to complete the sessions were sent to participants who did not complete the session within the designated 7 days. The content of experimental and control group procedures is provided in **Figure 2**. The content of the self-efficacy intervention and education condition was partially adapted from a previously developed internet-based intervention for survivors of direct exposure to trauma (Steinmetz et al., 2012).

The experimental group procedures (see **Figure 2**) included techniques which were complementary to face-to-face cognitive-behavior treatment, such as activity planning, skill training, and cognitive bias modification (cf. Eichenberg and Ott, 2012). The procedures used read- and write self-efficacy enhancing online exercises. The exercises referred to: identifying and recollecting one’s own mastery experience, analyzing personal experiences of dealing with barriers, planning for self-efficacy enhancement, identifying negative thoughts indicating self-doubts and transforming them into self-efficacy statements, and identifying positive emotions accompanying self-efficacy statements. The exercises required the participants to write their thoughts and statements online. Across the exercises, participants were asked to choose the context: they could refer to dealing with any stressors encountered at home or work. The elicited self-efficacy statements were contextualized respectively. They either referred to the work-related tasks and stressors, including indirect exposure to traumatic events or to home-related tasks and stressors. The homework assignments included suggestions about how participants might try to enhance their psychosocial resources (there were no specific homework assignments to be completed online).

The control group procedures (see **Figure 2**) included read-only educational materials, without exercises which required writing statements online. The education referred to resources that could enable workers to manage work-related tasks and work-related stress, including indirect exposure to traumatic events. The materials discussed various stressors (work-related and home-related), social and psychological resources (including social support and self-efficacy) that enable individuals to deal with stressors, and adverse consequences of stress at work, including STS and job burnout. The homework assignments included suggestions about how participants might try to enhance their psychosocial resources.

In an initial pilot study apart from the experimental and control group procedures we also developed active control group procedures which differed from the experimental group in terms of the enhanced cognitions/skills. Specifically, they focused on skills and beliefs referring to eliciting *social support* to deal with stressors. These procedures included read- and write online exercises and cognitive-behavioral treatment based techniques similar to those used in the experimental group. The initial feasibility study showed that participants of this active comparator group were not satisfied with the procedures as was indicated with 78% of enrolled professionals dropping out before the completion of four sessions. Therefore, the procedures of a second active control comparator group were not included in the present study.

### Measures

The descriptive statistics for all measures are provided in **Table 1**.

**Table 1:** Correlations between the study variables (*N* = 168).

Variable	1	2	3	4	5	6	7	8	9	10	11	*M (SD*)
(1) Self-efficacy T1	-											4.99 (0.81)
(2) Self-efficacy T2	0.53***	-										5.45 (0.71)
(3) Self-efficacy T3	0.44***	0.74***	-									5.54 (0.67)
(4) Secondary Traumatic Stress T1	-0.54***	-0.42***	-0.39***	-								2.33 (0.62)
(5) Secondary Traumatic Stress T2	-0.43***	-0.62***	-0.52***	0.70***	-							2.23 (0.57)
(6) Secondary Traumatic Stress T3	-0.23**	-0.48***	-0.59***	0.66***	0.67***	-						2.29 (0.59)
(7) Secondary Posttraumatic Growth T1	0.19*	0.34***	0.44***	-0.03	-0.16*	-0.01	-					2.89 (0.99)
(8) Secondary Posttraumatic Growth T2	0.09	0.32***	0.48***	-0.07	-0.18*	-0.16*	0.61***	-				3.10 (0.78)
(9) Secondary Posttraumatic Growth T3	0.03	0.32***	0.36***	0.00	-0.11	-0.07	0.69***	0.69***				3.06 (0.80)
(10) Age	0.10	0.09	0.10	-0.14	-0.12	-0.13	0.04	0.06	0.05			37.49 (10.39)
(11) Gender	-0.08	0.06	0.14	-0.06	-0.11	-0.13	0.13	0.13	0.14	-0.02		–
(12) Indirect exposure to trauma at T1	0.29***	0.09	0.10	-0.31***	-0.05	-0.22**	-0.11	-0.14	-0.14	-0.02	-0.07	2.63 (0.88)

*p < 0.05. **p < 0.01. ***p < 0.001. T1-measurement at Time 1 (before the intervention), T2-measurement at Time 2 (1-month after the baseline), T3-measurement at Time 3 (2-month follow up). Means represent a mean item response for a respective scale.

#### Secondary Traumatic Stress

The frequency of experiencing STS symptoms was measured with the Secondary Traumatic Stress Scale (STSS; Bride et al., 2004) at T1, T2, and T3. The scale consists of 17-items, measuring three areas of symptoms: intrusion, avoidance, and arousal. The responses are given on a scale ranging from 1 (*never*) to 5 (*very often*). The scale has good reliability, with Cronbach’s alphas obtained in the present study ranging from 0.90 at T1, 0.93 at T2, and 0.94 at T3.

#### Secondary Posttraumatic Growth

The Posttraumatic Growth Inventory—Short Form (PTGI–SF; Cann et al., 2010) was used to measure SPTG at T1, T2, and T3. Participants were asked to refer to growth-related experiences of indirect trauma exposure at work. The scale consists of 10 items that comprise five factors: spiritual change, appreciation of life, personal strength, relating to others, and new possibilities. The responses are provided on a scale ranging from 0 (*I did not experience this change as a result of my crisis*) to 5 (*I experienced this change to a very great degree as a result of my crisis*). The Cronbach’s alpha coefficients obtained in the present study were high, (0.90 at T1, 0.93 at T2, and 0.94 at T3).

#### Self-Efficacy

Secondary Trauma Self-Efficacy Scale (STSE; Cieslak et al., 2013b) was used to assess self-efficacy related to managing STS. The measure was applied at T1, T2, and T3. Participants were asked to provide their responses referring to the traumatic events that they indirectly experienced through their clients. An item example is “I am capable to cope with thoughts that I can’t handle working with these people anymore.” The scale has nine items, with responses ranging from 1 (*very incapable*) to 7 (*very capable*). In the present study, Cronbach’s alphas for the scale were 0.87 at T1 and 0.93 at both T2 and T3.

#### Indirect Exposure to Traumatic Events at Work

The Secondary Trauma Exposure Scale (Cieslak et al., 2013a) was applied at T1. This measure was designed to evaluate indirect exposure to traumatic events among healthcare providers. First, participants are asked to indicate if they experienced at least one of 10 types of events (e.g., natural disaster, sexual assault, military combat, and exposure to a war-zone) through their clients. All respondents declared indirect exposure to at least one traumatic event. Next, participants evaluated if these events, experienced *through their clients*, had affected them. The response scale ranged from 1 (*I was strongly affected by this event in a negative way*) through 3 (*I was affected by this even in a moderately negative way) to 7 (I was strongly affected by this event in a positive way*). Mean item response was 2.63 (*SD* = 0.88).

#### Demographic Variables

Participants completed background questions such as gender, age, years of work experience, education, and the type of profession.

### Analysis of Data

To investigate if the two study groups differed in terms of the outcomes (STS and SPTG), we completed an analysis of covariance, controlling the baseline levels of the respective variables and the index of indirect exposure at T1. The analyses were conducted to test the effects of the intervention on the outcomes (STS and SPTG) separately for T2, representing the measurement point at the completion of the intervention/control group procedures, and T3 (representing short-term follow up). The decision to test the effects at the post-test and the short-term follow-up separately was based on previous research documenting that the effects of short internet-based interventions on trauma-related outcomes may be different in size and the types of outcomes affected at post-tests, compared short-term follow-ups (Kuester et al., 2016). Bonferroni’s adjustment for multiple tests (four tests: two outcomes, two measurement points; assumed correlation between variables: *r*s > 0.08) lowered *p* levels to 0.014. As age and gender were unrelated to the outcomes (see **Table 1**) they were not controlled for in these analyses.

To investigate the indirect effects, that is to test whether the effects of the group assignment were mediated by self-efficacy, mediation analyses were performed using the PROCESS program, Model 4, with 10,000 bootstrapped replications (Hayes, 2013). PROCESS permits the conduct of mediator analysis in linear multiple regression models while accounting for the effects of covariates (T1 self-efficacy, and STS symptoms at T1 or SPTG at T1) on the mediator and the dependent variable. Similar procedures, assuming the mediating role of T2 self-efficacy—while controlling for the effects of T1 self-efficacy—were used in previous research investigating the mediating role of self-efficacy cognition between the group assignment and health outcomes (Luszczynska et al., 2016). As suggested by MacKinnon (2008), the independent variables, the mediators, and the dependent variables in the respective equations were measured at different time points (T1, T2, and T3) to establish temporal precedence. Thus, investigating the indirect effects with the outcomes evaluated at T2 would not allow for establishing the temporal precedence between the group assignment and the mediator or between the mediator and the outcome. Therefore, we chose to test the indirect effects only for the outcome measured at T3.

**FIGURE 3:**
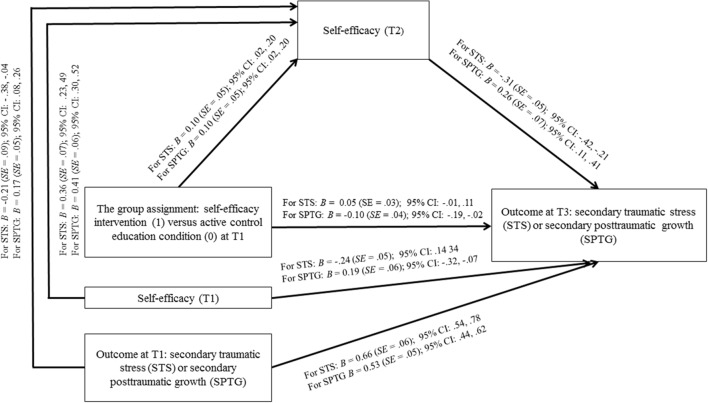
**Effects of the group assignment on secondary traumatic stress and secondary posttraumatic growth mediated by self-efficacy**.

Results of analyses are presented using two types of coefficients. The unstandardized regression coefficient (*B*) for each parameter is provided (see **Figure 3**). Furthermore, PROCESS estimates the indirect effect coefficient (𝜃) for each indirect pathway (through a mediator, T2 self-efficacy) between the independent variable (the group assignment) and the dependent variables (STS at T3 or SPTG at T3). The independent variables were coded as 1 (the experimental group with self-efficacy manipulation) or 0 (the control group).

Missing data were imputed with regression procedures (estimated maximization). In line with suggestions to apply intention–to–treat analysis for the experimental studies with health-related outcomes (Gupta, 2011), data from dropouts were also imputed. Missing data analysis indicated that data were missing completely at random, with Little’s χ*^2^* = (2035) = 1732.05, *p* = 1.00. Thus, the final analysis was conducted with a sample of *N* = 168.

## Results

### Preliminary Results

#### Attrition Analysis

Those who participated across three measurement points and completed all four experimental/control sessions (*n* = 68) were compared with *n* = 100 (59.5%) professionals who dropped out at any stage. Compared to completers, those who dropped out did not differ in self-efficacy at T1, *F*(1,166) = 2.23, *p* = 0.11, STS at T1, *F*(1,166) = 2.80, *p* = 0.10, SPTG at T1, *F*(1,166) = 1.66, *p* = 0.20, the indirect exposure to trauma at work, *F*(1,166) = 2.75, *p* = 0.10, gender, χ*^2^* = (1, *N* = 168) = 0.41, *p* = 0.52, age, *F*(1,158) = 0.95, *p* = 0.33, profession, χ*^2^* = (8, *N* = 165) = 3.11, *p* = 0.93), and the duration of employment, *F*(1,157) = 1.72, *p* = 0.19, η^2^ = 0.01. Finally, the dropout rates were the same for the experimental and the control groups, χ*^2^* = (1, *N* = 168) = 0.71, *p* = 0.40.

Those who dropped out were asked to provide reasons for not completing the study. The open–ended question was applied. Among those who responded (*n* = 54) the most frequent reasons to discontinue were personal reasons unrelated to the trial (39%) and the technical problems with the website or internet access (15%).

#### Randomization Check

Participants assigned to the two groups did not differ across the study variables. In particular, non-significant effects were found for age, *F*(1,166) = 0.95, *p* = 0.33, gender, χ*^2^* = (1, *N* = 168) = 0.46, *p* = 0.27, profession, χ*^2^* = (8, *N* = 165) = 4.40, *p* = 0.82, the duration of employment, *F*(1,166) = 0.09, *p* = 0.76, T1 indirect exposure, *F*(1,166) = 2.87, *p* = 0.09, self-efficacy at T1, *F*(1,166) = 2.53, *p* = 0.11, STS at T1, *F*(1,166) = 2.75, *p* = 0.10, and SPTG at T1, *F*(1,166) = 0.97, *p* = 0.33.

#### Associations among the Study Variables

The associations among the main variables of the study are presented in **Table 1**. Higher self-efficacy was related to lower STS symptoms across all three measurement points. Higher SPTG (T1) was associated with higher self-efficacy at all measurement points. SPTG at T2 and T3 was associated with self-efficacy at T2 and T3. There was no significant association between STS at T1 and SPTG at any time of measurement. However, a higher level of STS at T2 was associated with lower SPTG at T1 and T2.

Across the study variables, participants’ age was only significantly related to STS symptoms at T1, with older participants more likely to report higher levels of STS symptoms at T1 (*r* = 0.21, *p* = 0.01). For all other variables measured at T1, T2 or T3 the associations with age were negligible with all *r*s < 0.13, all *p*s > 0.11. Additionally, gender was unrelated to variables measured at T1, T2, or T3, all *F*s < 3.01, all *p*s > 0.08.

#### Effects on Secondary Trauma Self-Efficacy

The analyses of covariance (with T1 self-efficacy entered as the covariate) showed a significant effect of the group assignment on self-efficacy at T2, *F*(2,165) = 6.05, *p* = 0.015, η^2^ = 0.035. Overall, stronger T2 self-efficacy was observed in the self-efficacy intervention group (see **Table 2**). The effects were of medium size (**Table 2**). The effect of the group assignment on self-efficacy at T3 was not significant, *F*(2,165) = 2.24, *p* = 0.113, η^2^ = 0.015.

**Table 2:** Means, standard deviations, within-group and between-group effect sizes (Cohen’s *d*).

		*M (SD)*			Within-group *d* [95% CI]		Between-group *d* [95% CI] Education vs Self-efficacy	
Variable	Intervention	T1	T2	T3	Follow-up effect (T1–T2)	Short-term effect (T1–T3)	T2	T3
Self-efficacy	Education	4.89 (0.80)	5.29 (0.69)	5.43 (0.69)	-0.53 [-0.56, -0.23]	-0.72 [-0.71, -0.36]	-0.45 [-0.53, -0.10]	-0.33 [-0.42, -0.02]
	Self-efficacy	5.09 (0.81)	5.60 (0.70)	5.65 (0.65)	-0.67 [-0.67, -0.35]	-0.76 [-0.73, -0.39]		
	Total	4.99 (0.81)	5.45 (0.71)	5.54 (0.67)	-0.60 [-0.57, -0.34]	-0.74 [-0.67, -0.43]		
Secondary traumatic stress	Education	2.42 (0.66)	2.37 (0.58)	2.32 (0.68)	0.08 [-0.04, 0.14]	0.15 [-0.00, 0.19]	0.49 [0.09, 0.43]	0.08 [-0.13, 0.23]
	Self-efficacy	2.26 (0.58)	2.10 (0.52)	2.27 (0.51)	0.29 [0.05, 0.26]	-0.02 [-0.13, 0.11]		
	Total	2.33 (0.62)	2.23 (0.57)	2.29 (0.59)	0.17 [0.03, 0.17]	0.07 [-0.04, 0.12]		
Posttraumatic growth	Education	2.79 (0.98)	3.18 (0.77)	3.10 (0.79)	-0.44 [-0.54, -0.23]	-0.35 [-0.47, -0.15]	0.18 [-0.10, 0.38]	0.09 [-0.18, 0.31]
	Self-efficacy	2.99 (1.00)	3.04 (0.78)	3.03 (0.81)	-0.06 [-0.23, 0.13]	-0.04 [-0.20, 0.10]		
	Total	2.89 (0.99)	3.10 (0.78)	3.06 (0.80)	-0.24 [-0.33, -0.09]	-0.19 [-0.28, -0.06]		

*Education, education active control condition; Self-efficacy, Self-efficacy intervention condition; T1, baseline; T2, 1-month follow-up; T3, 2-month follow-up.*

### Effects of the Group Assignment on STS and SPTG

The first hypothesis assumed that compared to the control (read-only; education) condition, participants in the experimental (interactive; self-efficacy) condition would present lower STS and higher SPTG at 1-month follow-up (Time 2) and at 2-month follow-up (Time 3). Two sets of analyses of covariance were conducted to test this hypothesis, controlling for T1 measurement of respective variables. Descriptive statistics for all groups are displayed in **Table 2**. **Table 2** yields effect sizes (Cohen’s *d*s and their 95% Confidence Intervals).

#### Effects on Secondary Traumatic Stress

The analysis of covariance (with STS at T1 and indirect exposure included as the covariates) showed a significant effect of the group assignment on STS at T2, *F*(3,164) = 6.76, *p* = 0.010, η^2^ = 0.040. Overall, weaker STS symptoms (T2) were found in the self-efficacy intervention group (see **Table 2**). The observed effects were of medium size (**Table 2**). The effect of the group assignment on STS at T3 was not significant, *F*(3,164) = 0.52, *p* = 0.470, η^2^ = 0.003.

#### Effects on Secondary Posttraumatic Growth

Analysis of covariance (with T1 SPTG and the index of indirect exposure included as the covariates) yielded a significant effect of the group assignment on T2 SPTG, *F*(3,164) = 6.10 *p* = 0.013, η^2^ = 0.034. Overall, the control (education) group participants reported higher SPTG at T2 than the experimental group participants (**Table 2**). The observed effects were small (**Table 2**). The effect of the group assignment on SPTG at T3 was not significant, with *F*(3,164) = 3.54, *p* = 0.062, η^2^ = 0.021.

### Effects of the Group Assignment on STS and SPTG Mediated by Self-Efficacy: Results of Mediation Analyses

The second hypothesis indicated that the effects of the group assignment (control vs. experimental) would influence STS and SPTG at 2-month follow-up (Time 3) indirectly, with self-efficacy at Time 2 playing the mediating role. These effects were expected to occur after controlling for the values of self-efficacy and the respective outcome variable (either STS or SPTG) at the baseline (Time 1). Two mediation analyses, with STS and SPTG as respective outcomes, were conducted.

The first mediation analysis tested if the effects of the group assignment (T1) on the STS symptoms (at T3) may be mediated by self-efficacy at T2. The effects of the T1 level of the mediator (self-efficacy) and the T1 outcome (STS) on both the mediator (T2 self-efficacy) and the T3 outcome were controlled. The respective path coefficients are displayed in **Figure 3**. The experimental group assignment was related to higher T2 self-efficacy, which in turn was related to lower STS at T3 (see **Figure 3**). Overall, the variables included in the model explained 56% of STS at T3. The direct effect coefficient of the group assignment on T3 STS was not significant, *B* = 0.05 (*SE* = 0.03; 95% CI [-0.010, 0.114]). However, the indirect effect coefficient was significant, 𝜃 = -0.03, *SE* = 0.016 (95% CI [-0.071, -0.006]). These results suggested that although there was no direct effect of the intervention on T3 STS, the group assignment operated indirectly, via T2 self-efficacy.

The second mediation analysis tested if the effects of the group assignment (T1) on the SPTG (at T3) was mediated by self-efficacy at T2. The effects of T1 mediator (self-efficacy) and the T1 outcome (SPTG) on both the mediator (T2 self-efficacy) and the T3 outcome were controlled. The respective path coefficients are displayed in **Figure 3.** In particular, the experimental group assignment was related to higher T2 self-efficacy, which in turn was related to higher SPTG at T3 (**Figure 3**). Overall, the variables included in the model explained 53% of STS at T3. The direct effect coefficient of the group assignment on T3 SPTG was significant, *B* = -0.10 (*SE* = 0.04; 95% CI [-0.193, -0.021]). However, the indirect effect coefficient was also significant, 𝜃 = 0.03, *SE* = 0.01 (95% CI [0.006, 0.058]). These results suggested that the effects of the group assignment on T3 SPTG were mediated by T2 self-efficacy.

## Discussion

The findings of this study provide insight into the ways in which a self-efficacy intervention may affect mental health among professionals working with clients exposed to traumatic events. When we were not accounting for the underlying mediating processes, analyses of the changes in STS and SPTG suggested that the effects of the intervention on workers’ STS were observed only at 1-month follow-up (but not at 2-month follow-up), and that the direct influence on SPTG at 1-month follow-up was more pronounced in the active control education condition. However, mediation analyses showed that taking part in a self-efficacy enhancing intervention *indirectly* affected STS and SPTG at the 2-month follow-up, via its influence on self-efficacy. The more effective the intervention was regarding enhancing self-efficacy, the more likely it was that the participants reported lower STS symptoms and higher SPTG at 2-month follow-up.

The results of this experimental study are in line with social cognitive theory (Bandura, 1997) and research showing negative correlations between self-efficacy and employees’ mental health indicators, such as job burnout (Rogala et al., 2015) and symptoms of arousal, intrusion or avoidance (Luszczynska et al., 2009), and a positive relationship with SPTG (Shoji et al., 2014). Compared to an abundance of experimental research showing that self-efficacy interventions affect health behaviors through a mediator (e.g., a change in self-efficacy; Luszczynska and Schwarzer, 2015; cf. Luszczynska et al., 2016), we found no studies investigating if the effects of a self-efficacy intervention contributes to workers’ mental health via the mediating mechanism of self-efficacy change. Thus, the present study offers novel evidence and furthers the understanding of the mechanisms underlying the effects of self-efficacy interventions on positive and negative mental health outcomes.

Usually internet-based interventions targeting psychological consequences of the exposure to traumatic events focus on the symptoms of avoidance, intrusion, and arousal (Lange et al., 2000). Such interventions apply techniques which require that participants expose themselves to trauma reminders and engage in social sharing of trauma-related materials (Lange et al., 2000). In contrast, our intervention focused on enhancing participants’ optimistic beliefs about their ability to deal with a broad area of stressors and their consequences. This approach, based on social cognitive theory (Benight and Bandura, 2004), assumes that enhancing self-efficacy enables individuals to deal more effectively with a broad range of stressors, reinforces various optimistic beliefs and expectations, and, therefore, fosters positive mental health outcomes. Promotion of better mental health among workers indirectly exposed to trauma may require building up a broader range of resilience-related skills and beliefs (cf. Voss Horrell et al., 2011). Self-efficacy beliefs are considered the core resiliency beliefs (Benight and Cieslak, 2011). Self-efficacy facilitates dealing with a broad range of stressors, including those related to the exposure to trauma (Benight and Cieslak, 2011). Therefore, interventions enhancing self-efficacy may be used in both prevention and treatment programs for professionals exposed to various work-related stressors, including indirect exposure to trauma. Research should investigate further if self-efficacy interventions may affect a broader spectrum of mental health outcomes such job burnout or depression, but also other resilience-related cognitions such as optimism or ability to elicit social support.

The results shed some light on findings from a recent meta-analysis indicating that internet-based interventions aiming at a reduction of arousal, intrusion, and avoidance may produce effects similar in size to the effects of active control conditions (Kuester et al., 2016). These conclusions are drawn from analyses of the direct effects of the interventions on PTSD symptoms. However, the results of our study show that if the *underlying processes and mediating mechanisms* are taken into account, then significant indirect effects of the intervention on STS may be observed. Thus, the overall effects of our intervention were explained by its efficiency in changing self-efficacy cognitions. Future studies need to investigate which cognitive and behavior techniques may have the strongest effect on promoting self-efficacy.

The observed effects of the active control education-based condition provide support for the importance of education in promoting health and development of personal resources among health and human services professionals who are indirectly exposed to trauma (Newell and MacNeil, 2010). As suggested by Newell and MacNeil (2010) professional training for these workers should include information about indirect exposure, its consequences, and preventive individual resources. In the present study, professionals taking part in the education-based condition received this type of information. The education participation resulted in an increase of SPTG at 1-month follow-up (see **Table 2**).

The effects of the education-based control condition on SPTG highlighted the relevance of considering both positive and negative mental health outcomes when investigating the effects of psychosocial interventions. Studying positive and negative outcomes is in line with theoretical approaches and research showing the relevance of both positive and negative consequences of (direct or indirect) exposure to trauma (Arnold et al., 2005; Cann et al., 2010; Park, 2010). On the other hand, our findings provide no insight into the underlying mechanisms of the change observed in the education-based condition. For example, we cannot conclude whether the change in SPTG was mediated by the increase of knowledge on the consequences of exposure or if it was mediated by a change in skills to deal with stressors. As indicated by Roepke (2015), recent intervention studies provide no insight into the mediating mechanisms explaining a change in posttraumatic growth among trauma survivors. Unfortunately, this conclusion also applies to our study. Future research should consider testing for the mediating mechanisms explaining potential effects in the education-based control conditions in addition to the intervention group.

### Limitations, Future Directions, and Conclusion

Our study has several limitations. We observed a large dropout rate in both study conditions. Furthermore, the initial feasibility trial suggested that a potential active comparator control group procedure (focusing on enhancing skills referring to social support) resulted in a dramatic dropout of 78%. Strategies for imputing missing data have their limitations. High attrition may indicate that participants did not find the intervention sufficiently feasible and/or attractive. Future research should carefully evaluate the feasibility and attractiveness of the internet-based interventions targeting health service professionals for STS. The match between participants’ needs and the scope of the intervention was not evaluated. It is possible that targeting a broader range of cognitions and skills which enhance workers’ resilience to various work-related stressors could represent a better match and, therefore, result in better reach and better completion rates. Another major limitation refers to the use of short follow-ups. Effects of internet-based interventions targeting PTSD symptoms are significant when measured at post-tests, but often do not carry over at follow-ups (Kuester et al., 2016). As our study did not account for long-term follow-ups it is impossible to conclude about the effects of our intervention beyond 1 month after its completion. Furthermore, the intervention and control groups in the present study differed on two dimensions: the content (self-efficacy vs. education about stress and its consequences) and the interactivity (i.e., interactive exercises vs. read-only materials). This design does not allow for disentangling the effects of self-efficacy from the effects of the procedures applied enhancing these beliefs. In line with conclusions formulated by Kuester et al. (2016), we suggest that future research should investigate if the type of procedure has a moderating effect on the effectiveness of the intervention. We did not account for the T1–T3 changes in indirect exposure to trauma. An increase of the index of indirect exposure could affect such outcomes as STS or SPTG. Future research should account for this potential confounder. Finally, our study does not conform to all standards of fully randomized controlled trials, applying blinding procedures and evaluating the fidelity of the intervention processes. Thus, any conclusions should be treated with caution.

In sum, this study offers novel evidence and preliminary support for the effectiveness of a self-efficacy intervention for STS in health service professionals. The results have implications for practice, as brief self-efficacy interventions may be used in prevention and treatment of consequences of indirect trauma exposure among health and human services professionals. Although the direct effects of the intervention may not be significant at 2-month follow-up, the intervention exerted its influence indirectly, via self-efficacy beliefs. In particular, workers who took part in the intervention and due to the intervention experienced a self-efficacy enhancement, were more likely to report lower STS and higher SPTG at the 2-month follow-up.

## Author Contributions

RC conceived and designed the study, contributed to the statistical analysis, drafted and revised the manuscript; CB participated in the design and interpretation of the data and contributed to drafting the manuscript; AR participated in the design of the study, contributed to data collection and analysis, and contributed to drafting the manuscript; ES participated in the design of the study, contributed to data collection and analysis, and contributed to drafting the manuscript; MK participated in the design of the study, contributed to data collection, and contributed to drafting the manuscript; KZ participated in the design of the study, contributed to data collection, and contributed to drafting the manuscript; CY participated in the design and interpretation of the data and contributed to drafting the manuscript; AL participated in the design and interpretation of the data and contributed to drafting the manuscript.

## Conflict of Interest Statement

The authors declare that the research was conducted in the absence of any commercial or financial relationships that could be construed as a potential conflict of interest.
